# Effect of Roxadustat and Erythropoietin on Glycated Hemoglobin of Non-Dialysis Type 2 Diabetic Nephropathy Anemia Patients

**DOI:** 10.3390/biomedicines14040845

**Published:** 2026-04-08

**Authors:** Zhouxia Xiang, Wenqian Wei, Shunian Guo, Hanyu Meng, Shu Rong

**Affiliations:** Department of Nephrology, Shanghai General Hospital, Shanghai Jiao Tong University School of Medicine, Shanghai 200080, China

**Keywords:** diabetic nephropathy, Roxadustat, recombinant human erythropoietin, glycated hemoglobin, anemia

## Abstract

**Objectives**: To investigate the effects of Roxadustat and recombinant human erythropoietin (rHuEPO) on glycemic control and glycated hemoglobin (HbA1c) in non-dialysis type 2 diabetic kidney disease (DKD) patients with anemia. **Methods**: This retrospective study enrolled 449 patients, who were divided into three groups—the rHuEPO group (*n* = 252), the Roxadustat group (*n* = 102), and the switch group (*n* = 95)—in which patients were converted from rHuEPO to Roxadustat. All treatments lasted for more than three months. Changes in HbA1c and other indicators within groups as well as differences among groups were evaluated. **Results**: In the rHuEPO group, HbA1c levels decreased from 7.08 ± 1.19 to 6.41 ± 0.60 (*p* < 0.001), and they returned to baseline levels by 6–12 months (*p* > 0.05). In the Roxadustat group, HbA1c fluctuated but none of the differences reached statistical significance (*p* > 0.05). In the switch group, HbA1c decreased during rHuEPO treatment (*p* < 0.05) and returned to baseline after switching to Roxadustat (*p* > 0.05). No significant changes in blood glucose levels were observed in any group after treatment (*p* > 0.05). Multivariate linear regression analysis showed that changes in iron metabolism parameters, erythrocyte parameters, inflammatory markers, and glucose-lowering or lipid-lowering regimens had no significant effect on the change in HbA1c in the Roxadustat group (F = 0.834, *p* = 0.620), while the multivariate model of rHuEPO group also lacked statistical significance (F = 1.142, *p* = 0.170). After treatment, all three groups showed improvements in anemia, iron metabolism, renal function, inflammatory markers, and lipid profiles compared with baseline (*p* < 0.05). Additionally, further improvements in these parameters were observed after the transition from rHuEPO to Roxadustat (*p* < 0.05). Compared with rHuEPO group, the Roxadustat group exhibited significantly greater increases in hemoglobin, red blood cell count, total iron-binding capacity, transferrin, and serum iron (*p* < 0.05). **Conclusions**: In non-dialysis DKD patients with anemia, rHuEPO can significantly decrease HbA1c values, while Roxadustat does not. Roxadustat offers advantages over rHuEPO in terms of efficacy and assessment of glycemic control.

## 1. Introduction

Chronic kidney disease (CKD), which affects hundreds of millions worldwide, frequently leads to renal anemia (RA) due to reduced erythropoietin (EPO) production, disordered iron metabolism, and chronic inflammation-driven hemolysis; this burden is further exacerbated in patients with diabetic kidney disease (DKD), who experience earlier onset and greater severity of anemia than those with nondiabetic CKD [1,2]. Accurate glycemic monitoring is critical in this population. Glycated hemoglobin (HbA1c) is the standard measure of long-term glycemic control, reflecting average glucose levels over the preceding 2–3 months. However, HbA1c can be influenced by factors unrelated to glycemia, including erythrocyte turnover. Traditional erythropoiesis-stimulating agents (ESAs), widely used to treat RA, have been shown to lower HbA1c independently of blood glucose levels [3,4].

Hypoxia-inducible factor prolyl hydroxylase inhibitors (HIF-PHIs) represent a novel class of oral agents that stimulate endogenous EPO production by stabilizing hypoxia-inducible factors [5]. Roxadustat, the primary HIF-PHI used in China, has gained widespread use. While the overall efficacy and safety of Roxadustat in RA patients have been established, its effects on glycemic control remain unclear, particularly in the population transitioning from rHuEPO to Roxadustat. To address this gap, the present study investigates the effects of Roxadustat and rHuEPO on glycemic control indices and clinical efficacy, with a focus on HbA1c, in non-dialysis DKD patients with renal anemia.

## 2. Materials and Methods

### 2.1. Subjects

The study included patients with non-dialysis type 2 diabetes mellitus combined with CKD who were admitted to Shanghai General Hospital between December 2015 and December 2023. The study was approved by the Medical Ethics Committee of the General Hospital of Shanghai Jiao Tong University School of Medicine (No. 2025012).

Inclusion criteria: (1) Age ≥ 18 years. (2) Definite diagnosis of type 2 diabetes mellitus associated with proteinuria and/or renal function abnormalities: random urinary albumin/creatinine ratio ≥ 30 mg/g or urinary albumin excretion rate ≥ 30 mg/24 h, with values ≥ threshold in at least 2 of 3 repeat examinations conducted within 3–6 months before enrollment, or glomerular filtration rate (eGFR) < 60 mL/min·1.73 m^2^ for ≥3 months. (3) Diagnosis of RA and treatment with rHuEPO or Roxadustat.

Exclusion criteria: (1) Anemia caused by other diseases, such as thalassemia, sickle cell anemia, tumor-associated anemia, or myelodysplastic syndrome. (2) Recent (≤3 months) gastrointestinal bleeding and/or severe acute blood loss from other causes. (3) Anticipated initiation of renal replacement therapy within 24 weeks.

### 2.2. Groups

Enrolled patients were divided into three groups:

(1) Switch group included 95 patients who met the following criteria: (a) continuous rHuEPO treatment for >3 months; (b) an interval of <1 month between discontinuation of rHuEPO and initiation of Roxadustat; (c) continuous Roxadustat treatment for >3 months; (d) no change in hypoglycemic agents during the treatment period.
(2) rHuEPO group consisted of 252 patients who continued using rHuEPO exclusively for more than 3 months.
(3) Roxadustat group included 102 patients who received continuous treatment with Roxadustat for more than 3 months.

rHuEPO subcutaneous injection treatment started with an initial dose of 10,000 U once a week. The dose was then adjusted every 4 weeks based on the subject’s hemoglobin level to maintain a range of 100 to 120 g/L. Roxadustat capsule oral treatment began with a dose of 50 to 100 mg, 3 times a week, with each capsule containing 50 mg. The dose was adjusted every 4 weeks to maintain hemoglobin in the range of 100 to 120 g/L.

### 2.3. Data Collection

General data: Patient data included age, sex, body mass index (BMI), systolic and diastolic blood pressure, estimated glomerular filtration rate (eGFR), history of hypertension, cardiovascular and cerebrovascular diseases (myocardial infarction, cerebral infarction, cerebral hemorrhage, etc.), hyperglycemia, hypoglycemic agents (biguanides, sulfonylureas, glinides, DPP-4 inhibitors, α-glucosidase inhibitors, GLP-1 receptor agonists, SGLT2 inhibitors, insulin), lipid-lowering agents (statins, fibrates), blood pressure-lowering agents, and antihypertensive drugs (calcium channel blockers, renin–angiotensin system blockers, β-blockers, α1-blockers, diuretics, mineralocorticoid receptor antagonists).

Laboratory tests: hemoglobin (HB), red blood cell count (RBC), hematocrit (HCT), serum iron (SI), ferritin (SF), transferrin (TF), total iron-binding capacity (TIBC), blood urea nitrogen (BUN), blood creatinine (Scr), cystatin (Cys C), uric acid (UA), urine albumin creatinine ratio (UACR), total cholesterol (TC), low-density lipoprotein (LDL), high-density lipoprotein (HDL), serum calcium (Ca^2+^), C-reactive protein (CRP), interleukin 6 (IL-6), B-type natriuretic peptide (BNP), albumin (ALB), HbA1c, GLU. HbA1c was measured by high-performance liquid chromatography (HPLC) using a Sysmex HPLC system (Sysmex Corporation, Kobe, Japan), with results reported as a percentage (%). All laboratory tests were performed in the central clinical laboratory of Shanghai General Hospital following standard operating procedures.

Data collection time points: (a) Baseline: within 1 month before the first administration of rHuEPO (for the rHuEPO and switch groups) or Roxadustat (for the Roxadustat group). (b) Post-treatment: after 3 months (±1 week) of continuous treatment with rHuEPO, Roxadustat, or in the switch group.

### 2.4. Statistical Analysis

Statistical analyses were performed using SPSS 22.0 (IBM Corp., Armonk, NY, USA). Normality was assessed using the Shapiro–Wilk test. Continuous data are presented as mean ± standard deviation (SD) or median with interquartile range (IQR), as appropriate. Propensity score matching (PSM) was performed with R software (Version 4.3.1; R Foundation for Statistical Computing, Vienna, Austria) to address the potential confounding effect of unequal group sizes. A two-tailed *p* < 0.05 was considered statistically significant, unless otherwise specified after correction.

Paired or independent t-tests were used for normally distributed data, and Wilcoxon signed-rank or Mann–Whitney U tests for non-normally distributed data. The Friedman test with Bonferroni correction was applied for comparisons across multiple time points. Categorical variables were compared using the chi-squared test. Bonferroni correction was applied to control for multiple comparisons; the significance threshold was indicated in the footnotes.

Spearman correlation analysis was used to assess associations between changes in erythrocyte parameters and ΔHbA1c (‘Δ’ represents the changes before and after treatment). To evaluate the association between ΔHbA1c and potential confounding factors, multivariate linear regression analyses were performed separately in the rHuEPO and Roxadustat groups. ΔHbA1c was used as the dependent variable. The following covariates were included in the models: iron status (ΔSF, ΔSI, ΔTIBC, ΔTF), erythropoiesis rate (ΔHb, ΔHCT, ΔRBC), inflammatory markers (ΔCRP, ΔIL6), and concomitant medications. To avoid collinearity, glucose-lowering treatment regimens were categorized into three groups: oral agents alone, insulin alone, and combination therapy. Use of statins, fibrates, and iron supplements were recorded as binary variables (yes/no).

## 3. Results

### 3.1. Baseline

A total of 449 patients with non-dialysis DKD anemia were included, comprising 282 (62.8%) males and 167 (37.2%) females, with a mean age of 68.26 ± 12.34 years. No significant differences were observed among the three groups in baseline characteristics, including age, sex, BMI, systolic blood pressure, diastolic blood pressure, renal function, and history of cardiovascular disease, hypertension, or medication use (*p* > 0.05). These results are summarized in Table 1.

### 3.2. Comparison of Glycemic Control Indices Between rHuEPO and Roxadustat Treatment

#### 3.2.1. Overall Information

**Figure 1:**
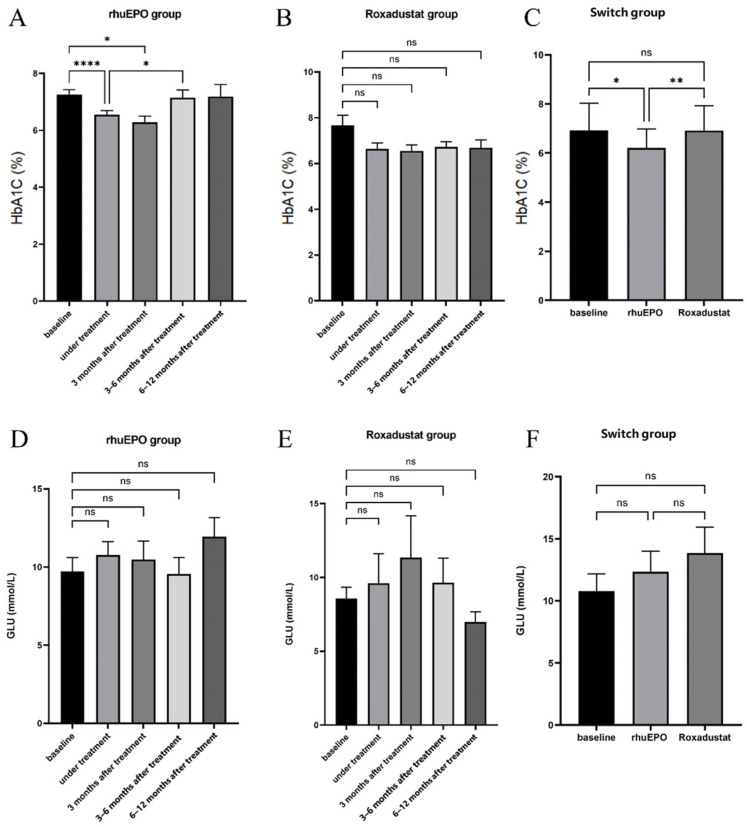
HbA1c and GLU changes in each group(**A**–**F**). Note: ns, *p* > 0.05; *, *p* < 0.05; **, *p* < 0.01; ****, *p* < 0.0001. (**A**) HbA1c changes in rHuEPO group; (**B**) HbA1c changes in Roxadustat group; (**C**) HbA1c changes in Switch group; (**D**) GLU changes in rHuEPO group; (**E**) GLU changes in Roxadustat group; (**F**) GLU changes in Switch group.

In the rHuEPO treatment group, a significant decrease in HbA1c levels was observed during the treatment period and at 3 months post-treatment (*p* < 0.001 and *p* < 0.05, respectively). HbA1c levels began to increase at 3–6 months post-treatment and returned to baseline levels at 6–12 months post-treatment. In the Roxadustat group, fluctuations in HbA1c levels were observed during the treatment period and up to 12 months post-treatment, but none of the differences were statistically significant (*p* > 0.05). The results are summarized in Figure 1 and Table 2.

A decrease in HbA1c during rHuEPO treatment was also observed in the switch group (*p* < 0.05). Interestingly, after switching to Roxadustat, HbA1c levels returned to baseline (*p* > 0.05). However, GLU levels did not change after treatment with either drug (*p* > 0.05). The results are shown in Figure 1 and Table 3.

#### 3.2.2. Propensity Score Matching Analysis

PSM was performed to adjust for differences in sample sizes across groups by R software. The matched analysis yielded results consistent with the unmatched analysis: although HbA1c levels fluctuated, no statistically significant differences were found between the Roxadustat groups (*p* > 0.05). See Supplementary Figure S1 for details.

Meanwhile, we selected patients from rHuEPO group at a 1:1 ratio to match baseline characteristics with those in the Roxadustat group (*n* = 102). The results from the PSM subset demonstrated that the trends in key parameters, such as HbA1c and Hb, were comparable to those derived from the original analysis, with no meaningful alterations in statistical significance. These findings are provided as Supplementary Table S1. Collectively, these results suggest that the main conclusions are robust despite the disparity in sample sizes between groups.

#### 3.2.3. Spearman Correlation Analysis

Spearman correlation analysis was performed to assess the associations between changes in erythrocyte parameters (ΔHb, ΔRBC, ΔHCT) as well as their percentage changes (ΔHb%, ΔRBC%, ΔHCT%) and ΔHbA1c. In the rHuEPO group, significant negative correlations were observed between ΔHbA1c and all erythrocyte parameters, including absolute changes (*p* < 0.001) and percentage changes (*p* < 0.001). In contrast, no significant correlations were observed in the Roxadustat group for either absolute or percentage changes (all *p* > 0.05). The results are shown in Table 4.

These findings suggest that the HbA1c-lowering effect of rHuEPO is closely associated with the rate of erythropoiesis, whereas Roxadustat does not exert a similar effect.

#### 3.2.4. Multivariate Linear Regression Analyses

Multivariate linear regression analyses were performed separately in the rHuEPO and Roxadustat groups to assess the associations between ΔHbA1c and potential confounding factors, including iron status, erythropoiesis rate, inflammatory markers, and concomitant medications. The results are summarized in Table 5.

In the rHuEPO group, the overall model was not statistically significant (F = 1.142, *p* = 0.170). ΔSI was significantly positively associated with ΔHbA1c (β = 0.063, 95% CI: 0.012 to 0.114, *p* = 0.017), and iron supplement use showed a borderline negative association (β = −0.680, 95% CI: −1.360 to 0.000, *p* = 0.050). No other variables reached statistical significance (all *p* > 0.05). In the Roxadustat group, none of the individual covariates were significantly associated with ΔHbA1c (all *p* > 0.05).

### 3.3. Comparison of Other Clinical Indicators Before and After Treatment in Three Groups

Compared with the pre-treatment period, anemia indicators (Hb, RBC, and HCT) increased in both the rHuEPO and Roxadustat groups. Iron metabolism indicators (SI, TIBC, and TF) increased, while SF levels decreased. Renal function indicators (BNP, BUN, Scr, Cys C, UA, and UACR) improved to some extent. Inflammatory markers (CRP and IL-6) decreased, and lipid parameters (TC and LDL) also decreased. Notably, the reduction in LDL was observed only in the Roxadustat group. These differences were statistically significant (all *p* < 0.05). In the switch group, significant changes in the above indicators (Hb, HCT, RBC, TIBC, SI, TF, BUN, Scr, UA, UACR, TC, CRP, and BNP) were confirmed after rHuEPO treatment compared with baseline, as shown in Table 3.

### 3.4. Comparison of Efficacy Between rHuEPO and Roxadustat Groups

No significant difference was found in pre-treatment laboratory values between the rHuEPO and Roxadustat groups (*p* > 0.05). Comparing the therapeutic effects of the two groups, after Roxadustat treatment, Hb, RBC, HCT, TIBC, TF, and SI increased more than in the rHuEPO group, with statistically significant differences (*p* < 0.0125). The reduction in indicators such as Scr, Cys C, LDL, and IL-6 were significantly greater in the Roxadustat group than in the rHuEPO group (*p* < 0.0125), as shown in Table 2.

### 3.5. Changes in Clinical Indicators After Switching from rHuEPO to Roxadustat in Switch Group

Compared with rHuEPO treatment, anemia indicators (Hb, RBC, HCT) and iron metabolism indicators (SI, TIBC, TF) further increased after switching to Roxadustat treatment. Cardiac and renal function indicators (BNP, BUN, Scr, UA, UACR) further improved. Inflammation indicators (CRP, IL-6) further decreased, and lipid indicators (TC, LDL) further decreased. These differences were statistically significant (*p* < 0.05). Notably, HDL increased after rHuEPO treatment compared with baseline, but the difference was not statistically significant. After switching to Roxadustat, HDL further increased with a statistically significant difference, as shown in Table 3.

## 4. Discussion

Consistent with previous reports that ESAs and iron supplementation can lower HbA1c levels independently of changes in blood glucose [4,6], the present study observed a significant reduction in HbA1c during rHuEPO treatment. Notably, this effect gradually diminished 3–6 months after treatment discontinuation, with HbA1c returning to baseline levels by 6–12 months. In contrast, Roxadustat did not significantly alter HbA1c levels, either during treatment or up to 12 months post-treatment. Furthermore, in patients switched from rHuEPO to Roxadustat, HbA1c levels returned to baseline after the transition, suggesting that the suppressive effect of rHuEPO on HbA1c is reversible and does not persist during Roxadustat therapy. Collectively, these findings indicate that Roxadustat does not interfere with HbA1c measurement, thereby offering an advantage in the accurate assessment of glycemic control in diabetic patients with CKD.

Roxadustat regulates and maintains erythropoiesis by stabilizing HIF activity in the body, and it has been shown to dose-dependently increase Hb levels and improve anemia and iron metabolism disorders in RA patients [5,7]. Clinical trials have demonstrated that Roxadustat has a favorable efficacy and safety profile compared to ESAs [8]. Additionally, it has been shown to effectively increase Hb, increase SI and TIBC, and decrease TF in both non-dialysis CKD and type 2 diabetes mellitus-combined CKD patients, with effects comparable to ESAs [8,9]. Our study is consistent with previous findings and found that anemia and iron metabolic status were further corrected by switching to Roxadustat treatment in patients previously treated effectively with rHuEPO.

Anemia in patients with type 2 diabetes and CKD is associated with increased risks of all-cause mortality, renal disease progression, and cardiovascular events [10,11]. Emerging evidence suggests that Roxadustat may confer cardiovascular and renal benefits beyond anemia correction. Previous clinical trials and a recent meta-analysis have shown that Roxadustat does not increase the risk of progression to end-stage renal disease and is associated with a lower incidence of composite cardiovascular endpoints compared with ESAs [12,13]. These benefits may be realized by HIF-1alpha [14]. In the kidney, HIF activation may ameliorate hypoxia, reduce inflammation, and attenuate tubular injury and interstitial fibrosis [15].

Consistent with these mechanistic insights, the present study demonstrated that both rHuEPO and Roxadustat significantly improved cardiac and renal function indices, with further improvements observed after switching from rHuEPO to Roxadustat. Additionally, Roxadustat treatment was associated with significant reductions in inflammatory markers (CRP, IL-6) and lipid parameters (TC, LDL). These findings align with previous reports suggesting that Roxadustat may exert anti-inflammatory and lipid-lowering effects, potentially contributing to its cardiorenal protective properties [16,17,18].

Several limitations of the present study should be considered when interpreting the findings: (1) The single-center, retrospective, non-randomized design is inherently susceptible to selection bias. Although PSM and multivariate regression analyses were performed, residual confounding resulting from unmeasured or imprecisely measured covariates (such as detailed nutritional status and red blood cell lifespan) cannot be entirely excluded. (2) The substantial heterogeneity in concomitant medications remains a potential source of residual confounding. (3) The relatively short follow-up period limits the evaluation of long-term renal, cardiovascular, and hard clinical endpoints. (4) Alternative glycemic markers, including glycated albumin and fructosamine, were not measured in this study; consequently, the interpretation of the observed changes in HbA1c should be regarded as preliminary. (5) Safety outcomes and adverse events occurring during the treatment and follow-up periods were not systematically assessed. To overcome these limitations and validate our findings, well-designed, multicenter, prospective studies with extended follow-up durations and comprehensive glycemic monitoring are warranted.

In conclusion, our findings confirmed that rHuEPO significantly decreased HbA1c values, while Roxadustat did not. Meanwhile, both rHuEPO and Roxadustat did not affect GLU levels while improving anemia, iron metabolism, renal function, inflammatory markers, and lipid profiles compared with baseline. Compared with rHuEPO, Roxadustat exhibited significantly greater improvement in Hb, HCT, RBC, TIBC, TF, and SI. Roxadustat offers advantages over rHuEPO in terms of efficacy and assessment of glycemic control.

## Figures and Tables

**Table 1:** Comparison of baseline information among the three groups.

	Age (Years)	BMI (kg/m^2^)	eGFR [mL/(min·1.73 m^2^)]	Male [n (%)]	Cardiovascular Diseases [n (%)]	Hypertension [n (%)]
Switch Group (*n* = 95)	68.23 ± 12.35	24.29 (22.49, 27.50)	31.45 ± 2.95	55 (57.9)	80 (84.2)	47 (49.5)
rHuEPO Group (*n* = 253)	68.45 ± 12.19	24.24 (22.38, 25.79)	29.73 ± 1.46	166 (65.6)	225 (88.9)	123 (48.6)
Roxadustat Group (*n* = 102)	67.78 ± 12.79	24.67 (23.61, 26.43)	30.09 ± 2.61	61 (59.8)	87 (85.3)	37 (36.0)
statistic	F = 0.11	H = 5.71	F = 0.12	χ^2^ = 2.22	χ^2^ = 1.76	χ^2^ = 5.04
*p* value	0.898	0.058	0.943	0.329	0.415	0.080
	cerebrovascular diseases	systolic blood pressure	diastolic blood pressure	Bisphosphonates	sulfonylureas	gliadinides
	[n (%)]	(mmHg)	(mmHg)	[n (%)]	[n (%)]	[n (%)]
Switch Group (*n* = 95)	31 (32.6)	147.80 ± 22.54	80.61 (72, 90)	10 (10.5)	15 (15.8)	40 (42.1)
rHuEPO Group (*n* = 253)	93 (36.8)	144.76 ± 20.21	79.24 (72, 85)	32 (12.6)	31 (12.3)	90 (35.6)
Roxadustat Group (*n* = 102)	28 (27.5)	147.42 ± 20.62	79.19 (75, 88)	15 (14.7)	14 (13.7)	31 (30.4)
statistic	χ^2^ = 2.89	F = 1.05	H = 3.24	χ^2^ = 0.78	χ^2^ = 0.77	χ^2^ = 2.95
*p* value	0.236	0.353	0.198	0.678	0.682	0.229
	DDP4 inhibitors	α-glucosidase inhibitors	GLP1 receptor agonists	SGLT2 inhibitors		Insulin
	[n (%)]	[n (%)]	[n (%)]	[n (%)]		[n (%)]
Switch Group (*n* = 95)	71 (74.7)	25 (26.3)	6 (6.3)	33 (34.7)		63 (66.3)
rHuEPO Group (*n* = 253)	180 (71.1)	77 (30.4)	21 (8.3)	78 (30.8)		183 (72.3)
Roxadustat Group (*n* = 102)	72 (70.6)	29 (28.4)	12 (11.8)	39 (38.2)		70 (68.6)
statistic	χ^2^ = 0.53	χ^2^ = 0.60	χ^2^ = 1.94	χ^2^ = 1.90		χ^2^ = 1.36
*p* value	0.767	0.742	0.379	0.387		0.508
	Statins	fibrates	calcium channel blockers	Renin–angiotensin system blockers		
	[n (%)]	[n (%)]	[n (%)]	[n (%)]		
Switch Group (*n* = 95)	72 (75.8)	21 (22.1)	87 (91.6)	66 (69.5)		
rHuEPO Group (*n* = 253)	182 (71.9)	37 (14.6)	227 (89.7)	167 (66.0)		
Roxadustat Group (*n* = 102)	65 (63.7)	16 (15.7)	84 (82.4)	63 (61.8)		
statistic	χ^2^ = 3.78	χ^2^ = 2.87	χ^2^ = 5.02	χ^2^ = 1.31		
*p* value	0.151	0.238	0.081	0.519		
	β-blockers	α1-blockers	diuretics	salicorticoid receptor antagonists		iron medicine
	[n (%)]	[n (%)]	[n (%)]	[n (%)]		[n (%)]
Switch Group (*n* = 95)	67 (70.5)	33 (34.7)	60 (63.2)	24 (25.3)		87 (91.6)
rHuEPO Group (*n* = 253)	163 (64.4)	61 (24.1)	148 (58.5)	42 (16.6)		231 (91.3)
Roxadustat Group (*n* = 102)	65 (63.7)	28 (27.5)	56 (54.9)	25 (24.5)		93 (91.2)
statistic	χ^2^ = 1.33	χ^2^ = 3.95	χ^2^ = 1.39	χ^2^ = 4.72		χ^2^ = 0.011
*p* value	0.513	0.138	0.499	0.095		0.995

Note: Bonferroni correction was applied for multiple comparisons; significance threshold *p* < 0.0167 (0.05/3).

**Table 2:** Comparison of indicators before and after treatment with rHuEPO and Roxadustat.

	rHuEPO Group			Roxadustat Group			*p* *
	Pre-Treatment	Post-Treatment	Difference in Value	Pre-Treatment	Post-Treatment	Difference in Value	
Hb (g/L)	76.86 ± 13.49	93.07 ± 12.69 ^a^	14.00 (9.00, 20.00)	81.41 ± 11.72	106.99 ± 12.67 ^a^	22.00 (14.00, 30.00)	<0.001
HCT (%)	23.40 (20.95, 23.43)	28.30 (27.00, 29.81) ^a^	6.50 (4.00, 9.00)	24.47 ± 3.62	32.26 ± 4.46 ^a^	8.50 (5.00, 13.00)	<0.001
RBC (×10^12^/L)	2.53 ± 0.45	3.54 ± 0.3 ^a^	0.95 (0.65, 1.30)	2.76 ± 0.48	4.24 ± 0.44 ^a^	1.40 (1.00, 1.85)	<0.001
TIBC (μmol/L)	37.48 ± 4.88	44.9 ± 5.2 ^a^	6.80 (3.50, 10.20)	38.38 ± 6.01	50.76 ± 7.21 ^a^	11.50 (7.00, 16.00)	<0.001
SI (μmol/L)	7.46 ± 2.98	12.69 ± 4.27 ^a^	4.90 (2.30, 7.80)	7.45 ± 2.41	16.17 ± 5.36 ^a^	8.00 (4.50, 12.00)	<0.001
SF (μg/L)	472.71 ± 249.83	170.79 ± 133.82 ^a^	−245.00 (−380.00, −150.00)	476.27 ± 231.72	222.29 ± 151.7 ^a^	−210.00 (−350.00, −120.00)	0.045
TF (g/L)	1.65 ± 0.34	2.15 ± 0.47 ^a^	0.45 (0.25, 0.68)	1.68 ± 0.39	1.75 (1.49, 1.78) ^a^	0.62 (0.35, 0.90)	<0.001
BUN (mmol/L)	28.37 ± 9.81	22.12 ± 7.9 ^a^	−5.20 (−10.00, −2.00)	28.59 ± 6.57	18.37 ± 4.33 ^a^	−8.50 (−13.00, −4.00)	<0.001
Scr (μmol/L)	303.95 (281.33, 326.57)	288.55 (262.59, 314.50) ^a^	−20.00 (−35.00, −8.00)	307.51 (271.14, 343.88)	285.72 (242.76, 328.67) ^a^	−32.00 (−48.00, −18.00)	<0.001
Cys C (mg/L)	2.22 ± 0.62	1.95 ± 0.63 ^a^	−0.22 (−0.50, 0.00)	2.16 ± 0.54	1.70 ± 0.40 ^a^	−0.38 (−0.65, −0.15)	<0.001
UA (μmol/L)	419.57 ± 91.46	375.11 ± 80.92 ^a^	−38.00 (−90.00, 10.00)	419.17 ± 86.14	364.9 ± 75.5 ^a^	−48.00 (−105.00, −5.00)	0.032
UACR (μg/mg)	728.61 ± 411.37	665.04 ± 314.23 ^b^	−45.00 (−180.00, 50.00)	728.37 ± 473	555.25 ± 340.2 ^b^	−120.00 (−300.00, −20.00)	<0.001
TC (mmol/L)	4.59 (4.39, 6.01)	4.55 (4.25, 5.63) ^a^	−0.30 (−0.80, 0.20)	4.92 ± 1.6	4.06 ± 0.73 ^a^	−0.70 (−1.30, −0.20)	<0.001
LDL (mmol/L)	2.29 (2.07, 2.69)	2.14 (1.93, 2.52)	−0.02 (−0.35, 0.30)	2.52 ± 0.94	2.13 ± 0.51 ^a^	−0.35 (−0.80, 0.00)	<0.001
HDL (mmol/L)	1.1 (1.02, 1.37)	1.16 (1.05, 1.21)	0.02 (−0.10, 0.15)	1.15 ± 0.31	1.17 ± 0.16	0.01 (−0.12, 0.14)	0.780
Ca^2+^ (mmol/L)	2.17 ± 0.23	2.18 ± 0.2	0.01 (−0.08, 0.10)	2.06 (2.04, 2.24)	2.20 (2.09, 2.19)	0.02 (−0.10, 0.12)	0.620
CRP (mg/L)	10.69 ± 8.33	9.26 ± 7.11 ^a^	−1.20 (−3.50, 1.00)	4.3 (6.02, 13.38)	7.2 (5.17, 10.48) ^b^	−2.00 (−5.00, 0.50)	0.018
IL6 (pg/mL)	11.76 (11.57, 29.35)	13.24 (9.72, 13.60) ^b^	−2.50 (−8.00, 3.00)	7.71 (8.13, 21.03)	10.77 (8.94, 11.99) ^b^	−5.00 (−12.00, 1.00)	0.009
BNP (pg/mL)	872 (658.22, 854.89)	598 (514.15, 718.15) ^a^	−40.00 (−120.00, 20.00)	563.94 ± 214.5	505.91 ± 164.28 ^b^	−55.00 (−150.00, −10.00)	0.021
ALB (g/L)	34.21 ± 6.33	34.09 ± 5.62	−0.10 (−2.50, 2.30)	34.3 ± 5.91	34.27 ± 5.12	−0.05 (−2.80, 2.50)	0.850
HbA1c (%)	7.08 ± 1.19	6.41 ± 0.60 ^a^	−0.65 (−1.10, −0.20)	7.67 (6.67, 8.68)	6.89 (6.38, 7.39)	−0.10 (−0.60, 0.40)	<0.001
GLU (mmol/L)	13.35 (7.81, 18.88)	12.62 (9.08, 16.15)	−0.50 (−3.50, 2.00)	8.6 (7.05, 10.15)	9.68 (5.56, 13.8)	−0.30 (−3.00, 2.50)	0.560

Note: ^a^, *p* < 0.001 compared with pre-treatment; ^b^, *p* < 0.05 compared with pre-treatment; *, difference in value of rHuEPO group VS. difference in value of Roxadustat group, with *p* values calculated using Mann–Whitney U test. Bonferroni correction applied for key comparisons; significance threshold *p* < 0.0125 (0.05/4).

**Table 3:** Comparison of switch group clinical data.

Indicators	Baseline	After rHuEPO Treatment	Roxadustat After Treatment
Hb (g/L)	83.61 ± 13.1	98.69 ± 11.7 ^a^	109.81 ± 12.85 ^ac^
HCT (%)	22.49 ± 3.46	28.37 ± 3.12 ^a^	33 (31.71, 37.74) ^ac^
RBC (×10^12^/L)	2.75 ± 0.4	3.38 (3.36, 3.63) ^a^	4.02 ± 0.31 ^ac^
TIBC (μmol/L)	38.58 ± 4.75	44.82 ± 4.88 ^a^	50.53 ± 5.04 ^ac^
SI (μmol/L)	8.45 ± 3.06	12.16 ± 3.93 ^a^	16.01 ± 5.3 ^ac^
SF (μg/L)	406.1 (388.05, 578.90)	219 (184.14, 291.10) ^a^	211.70 (140.89, 285.18) ^a^
TF (g/L)	1.69 ± 0.3	2.01 ± 0.41 ^a^	2.43 ± 0.5 ^ac^
BUN (mmol/L)	28.35 (24.76, 37.06)	23.36 ± 8.9 ^a^	19.14 ± 9.19 ^ad^
Scr (μmol/L)	308.53 ± 16.33	289.38 ± 11.88 ^a^	286.32 ± 15.61 ^ac^
Cys C (mg/L)	2.17 ± 0.6	1.94 ± 0.6	1.71 ± 0.69 ^a^
UA (μmol/L)	418.2 ± 84.58	378.73 ± 78.69 ^a^	368.49 ± 81.59 ^ac^
UACR (μg/mg)	806.45 (588.10, 977.75)	707.88 (596.97, 937.66) ^b^	547.57 ± 281.17 ^ac^
TC (mmol/L)	4.92 ± 1.33	4.74 ± 1.31 ^a^	4.1 ± 1.1 ^ac^
LDL (mmol/L)	2.49 ± 0.99	2.51 ± 0.87 ^a^	2.11 ± 0.6 ^ad^
HDL (mmol/L)	1.15 ± 0.33	1.16 (1.06, 1.21)	1.17 (1.08, 1.23) ^ac^
Ca^2+^ (mmol/L)	2.17 ± 0.24	2.18 ± 0.24	2.19 ± 0.2
CRP (mg/L)	11.19 ± 7.44	9.76 ± 6.71 ^a^	8.24 ± 6.38 ^ac^
IL6 (pg/mL)	14.94 ± 13.35	13.37 (9.21, 16.15)	10.7 (8.15, 11.70) ^bd^
BNP (pg/mL)	656.2 (390.65, 727.30)	530.1 ± 252.79 ^a^	506.3 (467.47, 591.81) ^ac^
ALB (g/L)	33.86 ± 6.28	33.71 ± 6.08	33.73 ± 5.75
HbA1c/%	6.95 ± 1.17	6.17 ± 0.78 ^a^	6.94 ± 1.07 ^c^
GLU (mmol/L)	11.75 (6.20, 17.3)	11.4 (6.17, 16.62)	13.01 (7.09, 18.93)

Note: ^a^, *p* < 0.001 compared with baseline; ^b^, *p* < 0.05 compared with baseline; ^c^, *p* < 0.001 compared with rHuEPO treatment period; ^d^, *p* < 0.05 compared with rHuEPO treatment period. Bonferroni correction applied for three-time-point comparisons; significance threshold *p* < 0.0167 (0.05/3).

**Table 4:** Spearman correlations between changes in erythrocyte parameters and change in HbA1c.

	rHuEPO Group		Roxadustat Group	
	rho(95% CI)	*p*	rho(95% CI)	*p*
ΔHb vs. ΔHbA1c	−0.477 (−0.602, −0.328)	*p* < 0.001	−0.091 (−0.289, 0.114)	0.376
ΔRBC vs. ΔHbA1c	−0.441 (−0.570, −0.289)	*p* < 0.001	−0.108 (−0.305, 0.097)	0.288
ΔHCT vs. ΔHbA1c	−0.452 (−0.580, −0.301)	*p* < 0.001	−0.086 (−0.284, 0.119)	0.399
ΔHb% vs. ΔHbA1c	−0.463 (−0.590, −0.313)	*p* < 0.001	−0.094 (−0.292, 0.111)	0.358
ΔRBC% vs. ΔHbA1c	−0.428 (−0.559, −0.274)	*p* < 0.001	−0.112 (−0.309, 0.093)	0.272
ΔHCT% vs. ΔHbA1c	−0.445 (−0.574, −0.293)	*p* < 0.001	−0.089 (−0.287, 0.116)	0.388

**Table 5:** Multivariate linear regression analyses of the rHuEPO and Roxadustat groups.

		β	95% CI	t	*p*	VIF
rHuEPO Group: F = 1.142, *p* = 0.170	ΔHb	0.011	(−0.016, 0.038)	0.809	0.421	1.251
	ΔHCT	0.037	(−0.020, 0.093)	1.288	0.202	1.08
	ΔRBC	−0.606	(−1.361, 0.148)	−1.605	0.113	1.319
	ΔTIBC	0.004	(−0.033, 0.042)	0.237	0.814	1.312
	ΔHb	−0.006	(−0.024, 0.012)	−0.649	0.519	1.125
	ΔSI	0.063	(0.012, 0.114)	2.457	0.017	1.631
	ΔSF	0.000	(−0.002, 0.002)	−0.152	0.880	1.303
	ΔTF	−0.379	(−0.886, 0.128)	−1.493	0.140	1.630
	ΔCRP	−0.003	(−0.029, 0.022)	−0.266	0.791	1.261
	ΔIL6	0.001	(−0.035, 0.037)	0.063	0.950	1.162
	antidiabetic drugs	−0.073	(−0.287, 0.141)	−0.679	0.500	1.136
	statins	−0.008	(−0.440, 0.423)	−0.039	0.969	1.214
	fibrates	0.339	(−0.094, 0.773)	1.562	0.123	1.373
	iron supplements	−0.68	(−1.360, 0.000)	−1.996	0.050	1.115
Roxadustat group: F = 0.834, *p* = 0.620	ΔHb	0.025	(−0.048, 0.098)	0.718	0.482	1.784
	ΔHCT	0.114	(−0.146, 0.374)	0.926	0.367	2.588
	ΔRBC	−7.307	(−25.591, 10.976)	−0.843	0.411	7.136
	ΔTIBC	0.018	(−0.101, 0.137)	0.320	0.753	1.729
	ΔSI	−0.046	(−0.230, 0.137)	−0.533	0.601	2.097
	ΔSF	0.003	(−0.003, 0.010)	1.041	0.312	1.958
	ΔTF	4.519	(−8.626, 17.665)	0.725	0.478	6.439
	ΔCRP	−0.015	(−0.178, 0.148)	−0.192	0.850	2.228
	ΔIL6	0.08	(−0.134, 0.294)	0.788	0.442	1.300
	antidiabetic drugs	−0.546	(−1.719, 0.627)	−0.983	0.340	1.842
	statins	0.864	(−0.810, 2.538)	1.089	0.291	1.353
	fibrates	−1.49	(−4.133, 1.152)	−1.190	0.250	1.655
	iron supplements	3.814	(−0.703, 8.332)	1.781	0.093	1.344

Note: Antidiabetic treatment categories: 1 = oral agents alone, 2 = insulin alone, 3 = combination therapy. Statins, fibrates, and iron supplements were included as binary variables (0 = no, 1 = yes).

## Data Availability

The raw data supporting the conclusions of this article will be made available by the authors on request.

## Supplementary Materials

The following supporting information can be downloaded at: https://www.mdpi.com/article/10.3390/biomedicines14040845/s1, Table S1: Comparison of indicators before and after treatment with rHuEPO and Roxadustat after PSM; Figure S1: HbA1c changes in each group after PSM.

## Abbreviations

The following abbreviations are used in this manuscript:

HbA1c	glycated hemoglobin
DKD	diabetic kidney disease
rHuEPO	recombinant human erythropoietin
PSM	propensity score matching
GLU	blood glucose
CKD	chronic kidney disease
RA	renal anemia
ESAs	erythropoiesis-stimulating agents
HIF-PHIs	hypoxia-inducible factor prolyl hydroxylase inhibitors
