# Supplementary material for: Effect of Roxadustat and Erythropoietin on Glycated Hemoglobin of Non-Dialysis Type 2 Diabetic Nephropathy Anemia Patients
Source: Biomedicines. 2026 Apr 8;14(4):845. doi: 10.3390/biomedicines14040845 (PMC13112930; doi:10.3390/biomedicines14040845)
Supplement: Supplementary file 1 [file biomedicines-14-00845-s001.zip › biomedicines-4201331-supplementary.pdf]

Supplementary Figure S1: HbA1c changes in each group after PSM

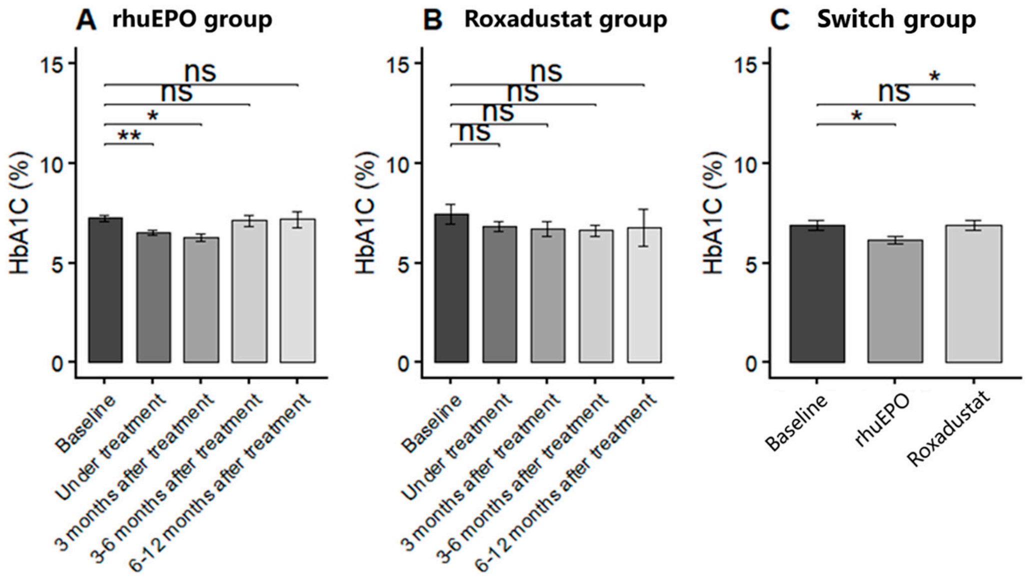

(A) HbA1c changes in rHuEPO group; (B) HbA1c changes in Roxadustat group;(C) HbA1c changes in Switch group. Note: ns, p > 0.05; *, p < 0.05; **, p < 0.01; ****, p < 0.0001.

Supplementary Table S1: Comparison of indicators before and after treatment with rHuEPO and Roxadustat after PSM

	rHuEPO Group-PSM			Roxadustat Group		
	pre-treatment	post-treatment	difference in value	pre-treatment	post-treatment	difference in value
Hb (g/L)	77.12 ± 12.86	93.45 ± 11.92[a]	16.33 (12.45, 19.87)	81.41±11.72	106.99±12.67[a]	25.57±10.92[c]
HCT (%)	23.45 (21.02, 23.48)	28.45 (27.12, 29.93) [a]	7.31 (5.89, 8.92)	24.47±3.62	32.26±4.46 [a]	7.80±3.83
RBC (×10^{12}/L)	2.55 ± 0.43	3.56 ± 0.29 [a]	1.01 (0.78, 1.23)	2.76±0.48	4.24±0.44 [a]	1.48±0.75 [c]
TIBC (μmol/L)	37.62 ± 4.76	45.08 ± 5.08 [a]	7.46 (5.12, 9.89)	38.38±6.01	50.76±7.21 [a]	12.37±7.36 [c]
SI (μmol/L)	7.52 ± 2.89	12.78 ± 4.15 [a]	5.26 (3.89, 6.78)	7.45±2.41	16.17±5.36 [a]	8.72±6.08 [c]
SF (μg/L)	475.23 ± 245.67	172.45 ± 131.28 [a]	-302.78 (-345.67, -258.34)	476.27±231.72	222.29±151.70 [a]	-253.97±202.25[d]
TF (g/L)	1.66 ± 0.32	2.17 ± 0.45 [a]	0.51 (0.32, 0.69)	1.68±0.39	1.75 (1.49, 1.78) [a]	0.08 (0.02, 0.19)[c]
BUN (mmol/L)	28.52 ± 9.68	22.25 ± 7.78 [a]	-6.27 (-9.89, -3.12)	28.59±6.57	18.37±4.33 [a]	-10.22±6.53 [c]
Scr (μmol/L)	305.12 (282.45, 327.89)	289.78 (263.45, 315.67) [a]	-24.35 (-32.45, -18.67)	307.51 (271.14, 343.88)	285.72 (242.76, 328.67) [a]	-79.72±16.51 [c]
Cys C (mg/L)	2.23 ± 0.61	1.96 ± 0.62 [a]	-0.27 (-0.45, -0.12)	2.16±0.54	1.70±0.40 [a]	-0.46±0.61 [d]
UA (μmol/L)	421.23 ± 90.12	376.45 ± 79.56 [a]	-44.78 (-67.89, -23.45)	419.17±86.14	364.90±75.50 [a]	-54.28±68.78
UACR (μg/mg)	730.45 ± 408.23	667.12 ± 311.45[b]	-63.33 (-189.45, 45.67)	728.37±473.00	555.25±340.20 [a]	-173.13±356.13 [c]
TC (mmol/L)	4.62 (4.41, 6.05)	4.58 (4.28, 5.68)[b]	-0.35 (-0.78, 0.12)	4.92±1.60	4.06±0.73 [a]	-0.86±1.32 [c]
LDL (mmol/L)	2.31 (2.09, 2.72)	2.16 (1.95, 2.55)	-0.02 (-0.34, 0.28)	2.52±0.94	2.13±0.51 [a]	-0.39±0.80 [c]
HDL (mmol/L)	1.11 (1.03, 1.38)	1.17 (1.06, 1.22)	0.03 (-0.23, 0.34)	1.15±0.31	1.17±0.16	0.02±0.27
Ca^{2+} (mmol/L)	2.18 ± 0.22	2.19 ± 0.19	0.01 (-0.12, 0.15)	2.06 (2.04, 2.24)	2.20 (2.09, 2.19) [a]	0.03±0.23
CRP (mg/L)	10.72 ± 8.21	9.31 ± 7.02[a]	-1.41 (-3.56, 0.67)	4.30 (6.02, 13.38)	7.20 (5.17, 10.48) [a]	-2.57±5.55
IL6 (pg/ml)	11.82 (11.62, 29.45)	13.28 (9.78, 13.65) [b]	-2.85 (-8.45, 2.34)	7.71 (8.13, 21.03)	10.77 (8.94, 11.99) [a]	-5.41±16.16
BNP (pg/ml)	875.23 (660.45, 857.12)	601.34 (516.23, 720.45)	-44.89 (-156.78, 45.23)	563.94±214.50	505.91±164.28 [a]	-58.03±125.94
ALB (g/L)	34.32 ± 6.25	34.18 ± 5.58	-0.14 (-2.45, 2.12)	34.30±5.91	34.27±5.12	-0.03±5.91
HbA1C (%)	7.10 ± 1.18	6.43 ± 0.59 [a]	-0.67 (-0.89, -0.45)	7.67 (6.67, 8.68)	6.89 (6.38, 7.39) [a]	-0.78±0.85 [c]
GLU (mmol/L)	13.42 (7.85, 18.92)	12.68 (9.12, 16.22)	-1.07 (-5.67, 3.45)	8.60 (7.05, 10.15)	9.68 (5.56, 13.80)	-1.02±9.50

Note: a, $P<0.001$ compared with pretreatment; b, $P<0.05$ compared with pretreatment; c, $P<0.001$ compared with rHuEPO group; d, $P<0.05$ compared with rHuEPO group. Bonferroni correction applied for key comparisons; significance threshold $P < 0.0125$ (0.05/4).
